# Pyrene-Functionalized Polyacetylenes: Synthesis and Photoluminescence Property

**DOI:** 10.3390/polym11081366

**Published:** 2019-08-19

**Authors:** Tanxiao Shen, Nan Jiang, Xiao’a Zhang, Lirong He, Xian Hua Lang, Jing Zhi Sun, Hui Zhao

**Affiliations:** 1 MOE Key Laboratory of Macromolecular Synthesis and Functionalization, Department of Polymer Science and Engineering, Zhejiang University, Hangzhou 310027, China; 2 Institute of Technical and Macromolecular Chemistry, University of Hamburg, Bundestrasse 45, 20146 Hamburg, Germany; 3 Institute of Fundamental and Frontier Sciences, University of Electronic Science and Technology of China, Chengdu 610051, China

**Keywords:** poly(diphenylacetylene), pyrene, excimer, interaction, energy transfer

## Abstract

Four pyrene-functionalized polyacetylenes were designed and prepared through a typical post-polymerization modification route, which is the highly efficient reaction between activated ester and primary anime groups. The chemical structures of the resultant polymers were characterized with multiple spectroscopic techniques and the data indicated the successful functionalization of the polyacetylenes. The introduction of the pyrene moieties into the polymer structure allowed us to investigate the interactions between the polymer backbone and side chains. For the mono-substituted polyacetylenes, both the monomer and excimer emission features of the pyrene groups could be recorded, while for the di-substituted polyacetylenes, the fluorescence from the pyrene excimer vanished and the fluorescence intensity from the pyrene monomer decreased, the fluorescence from the polymer chain predominated the emission features. The concomitant energy transfer from the pyrene monomer and excimer to poly(diphenylacetylene) backbone was associated with the underlying mechanism. In addition to the substitution modes, the linkage between the poly(diphenylacetylene) backbone and the pyrene moiety also played a significant role in the determination of the emission species. A long alkyl spacer was beneficial to the pyrene monomer emission while a short one may be helpful to the formation of the excimer and intramolecular energy transfer.

## 1. Introduction

Polyacetylenes play a unique role in the family of conjugated polymers and have received continuous attentions since the discovery of the metal conductivity in the highly doped solid films. Up to now, a variety of functional polyacetylenes have been synthesized and their properties have been investigated [1,2,3,4,5,6]. Generally, the functionalization of polyacetylene can be achieved by attaching a functional group to one side of the acetylene monomer (mono-substituted acetylene) or two functional groups to the two sides of the preset monomer (di-substituted acetylene), and then transforming the functionalized monomer into the corresponding polymer. Another strategy to synthesize functional polyacetylenes is the so-called post-polymerization modification (PPM) [7]. In this way, a functional polyacetylene can be derived from modifying a precursor polyacetylene with a specific functional group [7,8]. Taking the advantages of recent achievements in organic chemistry, highly efficient reactions in mild conditions such as amine-activated ester substitution [9,10], Cu(I) catalyzed azide-alkyne cycloaddition [11,12], Michael addition [13], phenol-yne addition [14] were engaged in the PPM route to prepare novel functionalized polyacetylenes. PPM is especially suitable for the functional poly(di-substituted acetylenes) bearing polar groups, which are unavailable from the direct polymerization route [15]. Through these routes, polyacetylenes have been bestowed various properties including liquid crystallinity [16], fluorescence emission [17], single-handed chirality [18], and circularly polarized luminescence [19].

Distinct from the functional polymers with saturated main-chains, where the main-chains serve for the system with inner matrix, mechanical strength and film formation ability, the main-chains in functional polyacetylenes are rigid and conjugated. In these aspects, the interactions between the polymer main-chains and the functional groups on the side chains show great impact on the performance of the whole system. For example, in a polyacetyelene-based side-chain liquid crystal polymer, the spacer linking the mesogen with the polymer backbone should be a long alkyl chain to render suitable flexibility and attenuate the rigidity of the conjugated polyene. Meanwhile, the phase transition points of the polyacetyelene-based liquid crystal are relatively higher as compared with the liquid crystal polymers with saturated main-chains [20,21,22,23]. For helical polyacetylenes, a hot research topic of alkyne-based functional polymers, the synergetic cooperation of main-chain rigidity and side-chain bulkiness allows to generate stable single-handed helicity [24,25,26,27]. The chirality transfer from the side chain to the main chain of a polyacetylene can be realized by the rational arrangement of a chiral center on the side chain to be nearing the main-chain or linking to the main chain via the rigid structure [28,29,30,31,32,33]. 

**Figure 1:**
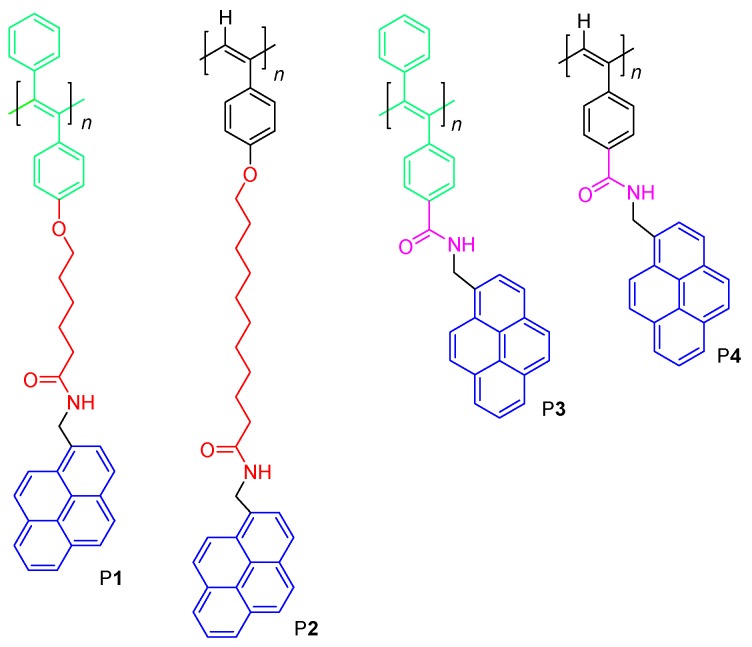
The chemical structures of the pyrene-functionalized polyacetylenes in this work.

In the above-mentioned studies, the research efforts are mainly focused on the spatially structural factors such as the bulky size, the length and flexibility of the side chains, the rigidity of the backbones. The understanding of the electronic interaction between the functional groups on the side chains and the main chains of polyacetylenes has received little attention though it is fundamentally important. Herein, we report our design, synthesis and photophysical investigation of a series of pyrene-functionalized polyacetylene derivatives. As displayed in Figure 1, pyrene is used as the functional group to modify the polyacetylenes, considering that pyrene has characteristic fluorescence features from the monomer and excimer emissions. This particularity makes pyrene moieties suitable for fluorescently probing the interactions between the main- and side-chains of the modified polymers [34,35,36,37]. Meanwhile, poly(phenylacetylene) and poly(diphenylacetylene) are chosen as polymer main chains, since the former and the latter are non-emissive and highly emissive, respectively. This distinction enables us to recognize the role of the polymer main chain. The linkages between the pyrene group and polymer main chain are a semi-rigid amide group and a flexible alkyl chain. This design is reasonable because the excimer formation between pyrene groups and electronic interaction between the pyrene groups and conjugated main chains depend on both spatial arrangement and distance.

## 2. Materials and Methods

### 2.1. Materials

Toluene and dichloromethane (DCM) were distilled under normal pressure over calcium hydride under nitrogen before use. Tetrahydrofuran (THF) was distilled under normal pressure from sodium benzophenone ketyl under nitrogen immediately prior to use. Triethylamine (Et_3_N) was distilled and dried over potassium hydroxide. Other solvents, including *N*,*N*-dimethyl- formamide (DMF), chloroform (CHCl_3_), methanol, ethyl acetate, hexane and petroleum ether (PE, b. p. 60–90 °C) were purchased from Sinopharm Co. Ltd (Hangzhou, China). They were of analytical grade and directly used as received without further purification. WCl_6_, Ph_4_Sn, CuI, PPh_3_ and [PdCl_2_⋅(PPh_3_)_2_] were bought from Aldrich (Shanghai, China). *N*,*N*′-dicyclohexylcarbodiimide (DCC), *p*-toluene sulfonic acid monohydrate (TsOH), 4-(dimethylamino)pyridine (DMAP), phenylacetylene, pyrene-methylamine, and pentafluorophenol were purchased from Acros (Shanghai, Chnia). Methyl 6-(4-iodo-phenoxy)hexanoate was obtained accroding to the procedures reported elsewhere [37].

### 2.2. Methods

The FTIR spectra were recorded on a Bruker Vector 22 FT-IR spectrophotometer (Hangzhou, China) by using thin films on KBr pellets. The ^1^H and ^13^C NMR spectra were recorded on a Bruker ARX 500 NMR spectrometer or Bruker ARX 400 (Hangzhou, China) in chloroform-*d* (or methanol-*d* and DMSO-*d*6) using tetramethylsilane (TMS; δ = 0 ppm) as an internal standard. Molecular weights (*M*_w_, *M*_n_) and polydispersity indexes (PDI, *M*_w_/*M*_n_) of the polymers were estimated by using a Waters PL-GPC-50 gel permeation chromatography (GPC) system equipped with a refractive index (RI) detector (Hangzhou, China). A set of monodisperse polystyrene standards covering the molecular weight range of 10^3^–10^7^ were used for molecular weight calibration. The centrifugation process was done on the Siemensstr 25 D-78564 Z-326 type centrifugal machine (Hangzhou, China) The thermogravimetric analysis (TGA) was conducted on a Pyris 6 thermogravimetric analyzer (Perkin-Elmer, Hangzhou, China) under N_2_ atmosphere at a heating rate of 20 °C·min^−1^. The UV–Vis absorption spectra and fluorescence spectra were recorded on a Varian CARY 100 Bio UV–Vis spectrophotometer (Hangzhou, China) and a Shimadzu RF-5301PC spectro-fluorophotometer (Hangzhou, China). 

## 3. Results

### 3.1. Polymer Synthesis

**Scheme 1:**
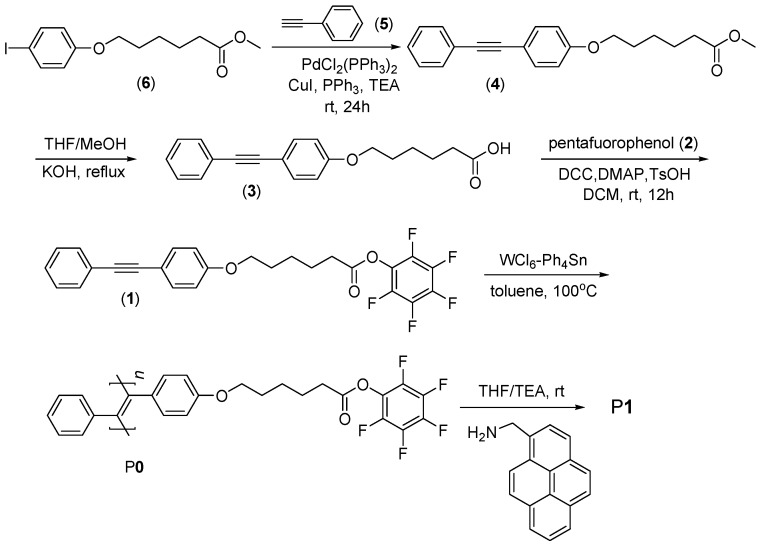
Synthetic route to a pyrene-functionalized poly(diphenylacetylene) (P**1**) from the precursor polymer (P**0**) with the post-polymerization modification strategy.

All of the target polyacetylene derivatives (P**1** to P**4**, Figure 1) were prepared through the PPM route. The details of the synthetic procedures and structure characterization data are described in Supplementary Materials (SM, Schemes S1–S3). Except for P**1**, the synthesis of the other three polymers P**2**, P**3**, and P**4** was conducted by the reaction of 1-aminomethylpyrene and corresponding polyacetylene precursors, which had been reported in our previous works [10,13,37,38,39,40,41]. The synthetic route to P**1** is shown in Scheme 1 and the experimental procedures are briefly introduced as follows. Sonogashira coupling between methyl 6-(4-iodophenoxy)hexanoate (**6**) and phenylacetylene (**5**), de-protection of the methyl hexanoate of the coupling resultant (**4**), and the esterification between the generated carboxylic acid (**3**) and pentafluorophenol (**2**) with the DCC catalyst were carried out step-by-step. The reactions went on smoothly and the yields of the intermediates and monomer **1** were good (ref. SM). WCl_6_-Ph_4_Sn was used as the complex catalyst to initiate the polymerization of **1** because this catalyst system has been successfully used to polymerize a series of disubstituted acetylene monomers bearing ester and activated ester groups [7,8,15]. Under a primitively optimized reaction condition (see SM), the precursor polymer P**0** with an average molecular weight of 13,400 (PDI =2.13) was obtained with a yield of 61.2% (Table 1). 

The transformation of P**0** to P**1** was a typical post-polymerization modification reaction. It has been well-documented that the carboxylic pentafluorophenol ester (activated ester) can be easily and completely substituted by stronger nucleophilic reagents such as primary and secondary amines, this strategy has been successfully adapted to the preparation of functional polyacetylenes. The expected polymers P**1** to P**4** were derived from the corresponding precursors by substitution of the pentafluorophenol with pyrene-methylamine. The results are summarized in Table 1, the yield of the final product could be as high as 95.60%. 

### 3.2. Structure Characterization

**Figure 2:**
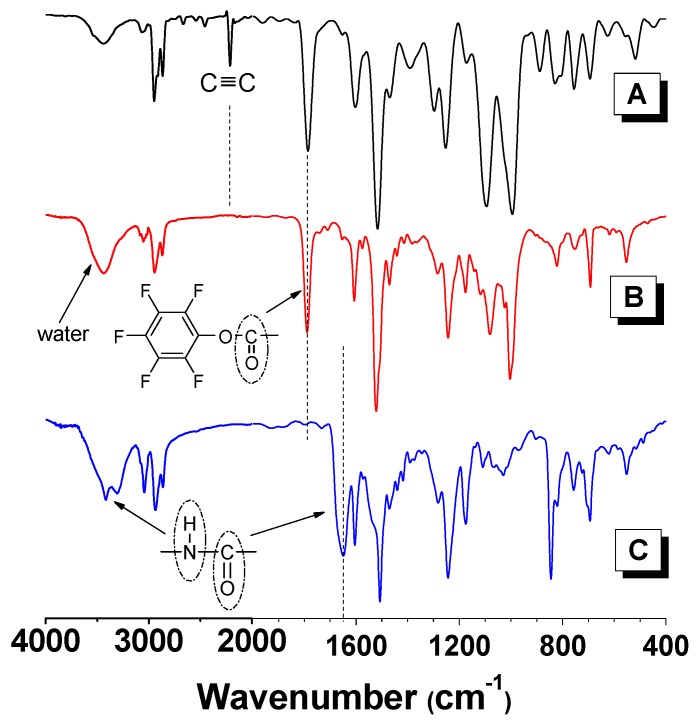
FTIR spectra of (**A**) monomer **1**, (**B**) precursor P**0** bearing activated ester groups, and (**C**) pyrene-functionalized poly(diphenylacetylene) derivative P**1**.

The chemical structure of the final products and the intermediates were characterized with multiple spectroscopic techniques and the data matched the expected structures. Here, we show the results by using P**1** and its precursor as representatives. Figure 2 displays the FTIR spectra of P**1**, its precursor P**0** and monomer **1**. The absorption band at 2216 cm^−1^ is assigned to the stretching vibration of C≡C, it appears in the spectrum of monomer **1** but disappears in the spectrum of P**0**. This change indicates that the C≡C bonds have been consumed in the polymerization reaction of monomer **1**. The vibration band at around 1786 cm^−1^ is ascribed to the stretching mode of the C=O (carbonyl) bond in an ester group. It remains almost unchanged in spectra A and B, suggesting the retention of the activated ester group in the polymerization process. In contrast, this band is vanished and a new band at around 1655 cm^−1^ emerges in spectrum C, which originates from the stretching mode of the C=O bond in an amide group. Concomitantly, a broad and intense absorption band covering 3300~3330 cm^−1^, corresponding to the vibration of the N–H bonds on amide group, also appears in spectrum C. This characteristic change indicates that the ester has been transformed to the amide group in the post-polymerization modification reaction. 

**Figure 3:**
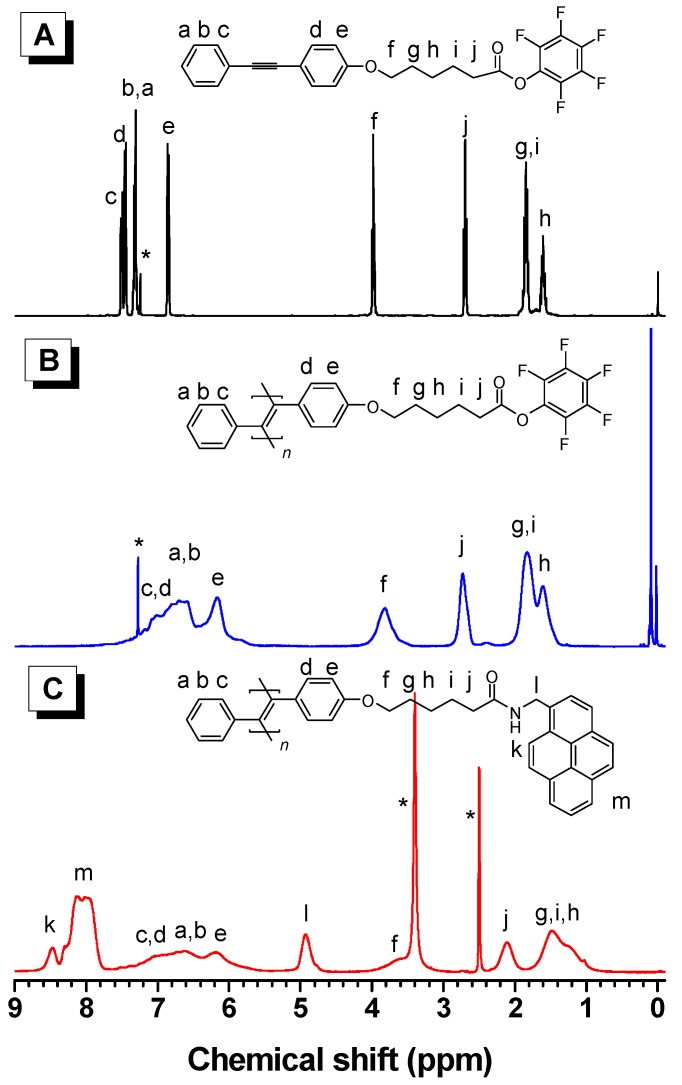
^1^H NMR spectra of (**A**) monomer **1** (in chloroform-*d*), (**B**) precursor P**0** bearing activated ester groups (in chloroform-*d*), and (**C**) pyrene-functionalized poly(diphenylacetylene) derivative P**1** (in DMSO-*d*6). The solvent and water peaks are marked with asterisks.

The ^1^H NMR spectra of monomer **1**, precursor polymer P**0** and the final pyrene-functionalized poly(diphenylacetylene) derivative P**1** are shown in Figure 3. The resonant peaks at around *δ*~7.50, 7.45, 7.30 and 6.86 ppm are assigned to the protons on the phenyl groups at the two sides of the C≡C bond (protons *a*~*e*, Figure 3A). These peaks shift to the high-field around *δ*~7.08–6.59 and 6.17 ppm in Figure 3B, which suggest the formation of the polymer chain due to the better conjugation of the phenyl groups and the polyene-backbone. Moreover, the peaks belonging to the protons on the phenyl groups, new peaks appear at around *δ*~8.31–7.80 ppm (*m*) in Figure 3C, which can be assigned to the protons on pyrene moiety. Meanwhile, a separated peak (*k*) appears at *δ*~8.47 ppm and it can be assigned to the singlet proton on the amide group. The new peak at *δ*~4.93 ppm (*l*) is then assigned to the methylene group between the pyrene and amide groups. It is noticeable that broader peaks were observed upon polymerizing the monomer M**0** to P**0**, which is a clear result of the much slower tumbling experienced by the protons bound to the rigid polyacetylene backbone. When P**0** was labelled with 1-pyrenemethyl amine the resonance peaks became broad again, indicating that the bulky pyrene and the stiff amide bond that was generated slowed down the tumbling of the polymer protons further. The structural rigidity affected the emission of the derived pyrene-functionalized polyacetylenes.

**Figure 4:**
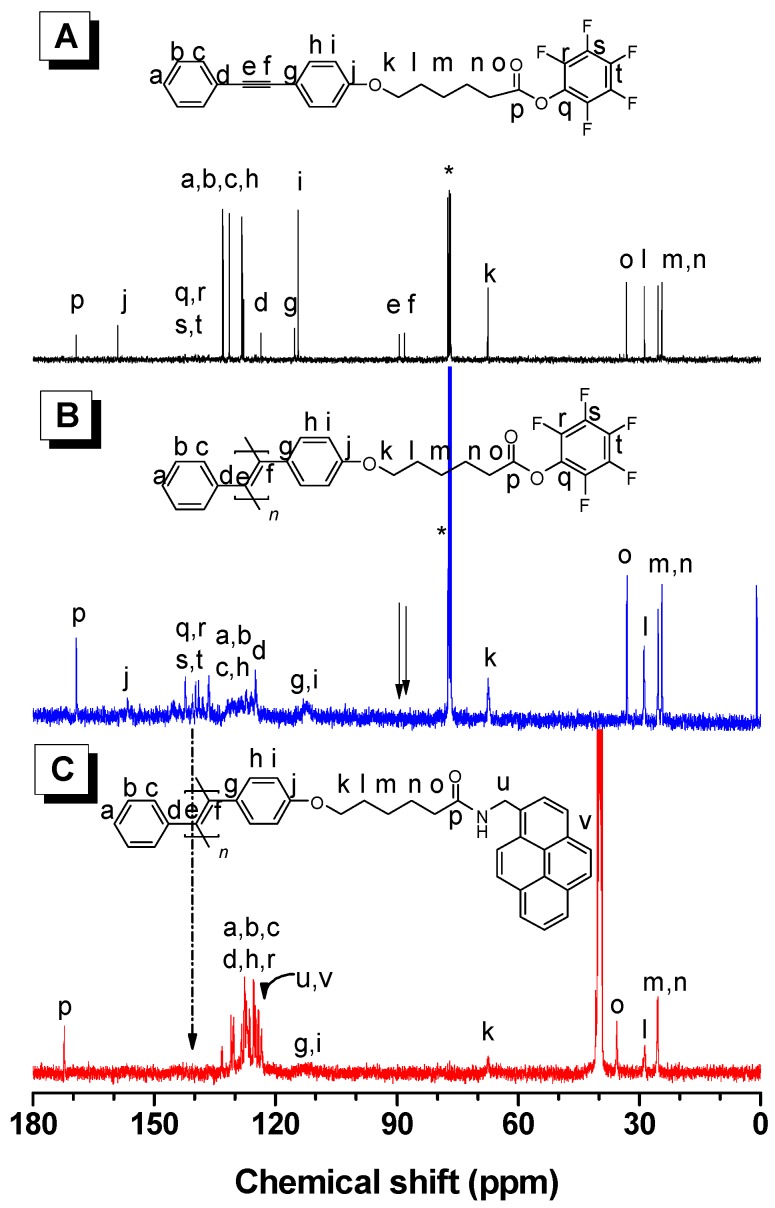
^13^C NMR spectra of (**A**) monomer **1** (in chloroform-*d*), (**B**) precursor P**0** bearing activated ester groups (in chloroform-*d*), and (**C**) pyrene-functionalized poly(diphenylacetylene) derivative P**1** (in DMSO-*d*6). The solvent and water peaks are marked with asterisks.

The ^13^C NMR spectra of monomer **1**, precursor polymer P**0** and the pyrene-functionalized poly(diphenylacetylene) P**1** present further proofs to confirm the formation of the expected structure (Figure 4). In Figure 4A, the peaks at δ~89.4 and 88.0 ppm originate from the two carbons on a C≡C bond. They disappear on the spectrum of P**0** (downwards arrows, Figure 4B), suggesting the complete consumption of the C≡C bonds in the polymerization reaction. After the modification reaction, the peaks at δ~123–128 ppm (u and v in C) can be observed while those at δ~133–140 ppm (q, r, s, and t in B) disappeared, these changes are inconsistent with the presence of the pyrene group and the absence of the pentafluorophenol group. All of the spectroscopic results confirmed that the designed pyrene-functionalized polyacetylenes have been successfully derived.

### 3.3. Absorption and Emission Behaviors

**Figure 5:**
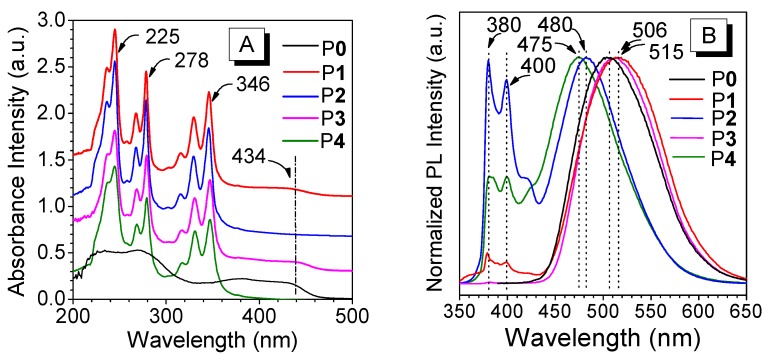
UV–Visible absorption (**A**) and normalized photoluminescence (PL) spectra (**B**) of the precursor polymer (P**0**) and the derived pyrene-functionalized Polyacetylenes (P**1**–P**4**). Solvent: THF, concentration: 0.02 mmol/L, λ_ex_ = 343 nm.

The UV–Visible absorption and photoluminescence emission spectra of the derived polyacetylenes have been measured and the data are shown in Figure 5A,B, respectively. The precursor polyacetylene (P**0**) has a broad absorption band covering the spectral range from 220 to 470 nm. Compared with the absorption spectrum of monomer **1** (see Figure S1), the weak absorption feature extending to 470 nm (a shoulder at around 430 nm) can be associated with the main chain of poly(diphenylacetylene). After the activated ester groups are substituted by pyrene-methylamine functionalities, a group of strong absorption bands emerge with main absorption peaks at 225, 278 and 346 nm, which are the characteristic absorption bands of the pyrene moiety, indicating the presence of the pyrene in the obtained polymer P**1**. The same absorption features can be observed for other three pyrene-modified polyacetylenes P**2**, P**3** and P**4**. The difference in the absorption feature of the mono- and di-substituted polyacetylenes is the presence and absence of the absorption tail with a shoulder at about 434 nm for poly(diphenylacetylenes) and poly(phenylacetylenes), respectively.

The photoluminescence (PL) spectra of these polymers in 0.02 mmol/L THF solutions were measured and the results are shown in Figure 5B. On the PL spectrum of the precursor polymer P**0**, a single and strong emission band peaked at 506 nm is observed (black line). After the substitution of the activated ester by pyrene-methylamine groups, the pyrene-functionalized polymer P**1** exhibits a different PL spectrum from P**0**. A strong emission band centered at around 515 nm is recorded and a weak emission feature with two sharp peaks at 380 and 400 nm is also recorded (red line). For the pyrene-functionalized mono-substituted polyacetylene P**2**, there are two emission bands on its PL spectrum, one is a broad band with a peak at around 480 nm without fine structure, and the other has two recognizable peaks at 380 and 400 nm as well as a shoulder peak at around 420 nm (blue line). When the pyrene groups are linked to the poly(diphenylacetylene) backbone, the derived polymer P**3** shows a predominately strong emission band with a maximum at around 515 nm and a very weak band (see the amplified spectra in Figure S2) in the UV spectral region (magenta line). On the PL spectrum of P**4**, the emission band with a maximum at around 475 nm is the dominant feature and a weak band whose features are similar to that observed for P**2** is present (olive line).

## 4. Discussion

The PL spectra of these pyrene-functionalized Polyacetylenes (P**1** to P**4**), in contrast to their absorption spectra, show evident distinctions. In order to clearly analyze and understand their emission properties, the PL spectrum of pyrene-methylamine was measured in the condition that is identical to the PL measurements of the polyacetylenes and the data are depicted as Figure S3. As shown in Figure 5B, a single and strong emission band peaked at 506 nm is observed for P**0** (black line) and this emission band can be associated with the fluorescence of poly(diphenylacetylene) backbone, which is consistent with the previously reported poly(diphenylacetylene) derivatives [10,11,12,13,14,15]. For pyrene-methylamine in 0.02 mmol/L THF solution, there are two emission bands, a featureless band peaked at 480 nm and a multiply-peaked band with the lowest energy peak at around 400 nm, which are assigned to the excimer and monomer emission bands of pyrene, respectively (Figure S3) [36,37,38,39,42]. Now, there are three emission bands, i.e. the poly(diphenylacetylene) backbone, monomer and excimer of pyrene groups, to be used for estimating the electronic interactions between the conjugated main-chain and functional groups on side chains. Comparing the PL features of P**1** with P**2** (Figure 5B), the strong emission band peaked at around 515 nm on the spectral line of P**1** (red line) can be assigned to the emission from the poly(diphenylacetylene) backbone; and the weak band with a maximum at 400 nm can be ascribed to the emission from the pyrene monomers. While the single-peaked band and the triple-peaked band on the spectral line of P**2** (blue line) are assigned to the emissions from the excimer and monomer of pyrene moieties. It can be seen that, for P**1**, the excimer emission of the pyrene moieties disappear and meanwhile the monomer emission becomes quite weak.

Where have the fluorescence from the pyrene excimers and monomers gone in P**1** THF solution? According to the chemical structure, the essential difference between P**1** and P**2** is the polymer backbone, which determines the emissive poly(diphenylacetylene) backbone of P**1** and non-emissive poly(phenylacetylene) backbone of P**2**. The energy transfer from pyrene to P**1** is a possible mechanism, since the emission band of poly(diphenylacetylene) (λ_em_~515 nm) in P**1** localizes in lower energy region as compared with the emission bands of pyrene excimers (λ_em_~480 nm) and monomers (λ_em_~400 nm). To verify this assumption, P**3**, an analogous polymer of P**1** was designed and prepared. The alkyl spacer between the pyrene group and poly(diphenylacetylene) backbone is cut off thereby the distance between the two parts becomes smaller. In principle, the energy transfer process depends on the spatial interval of the donor and acceptor, thus the energy transfer in P**3** must be more efficient than in P**1** under the same experimental conditions. This inference is tentatively validated by the PL spectrum of P**3** in the THF solution (magenta line, Figure 5B), both of the excimer and monomer emission bands are drastically quenched, only the trace amount of the monomer’s emission can be observed on the amplified spectrum (Figure S2). The observation of the monomer emission quenching may be also correlated with the facile formation of the excimers in P**3**, which will be discussed later.

**Figure 6:**
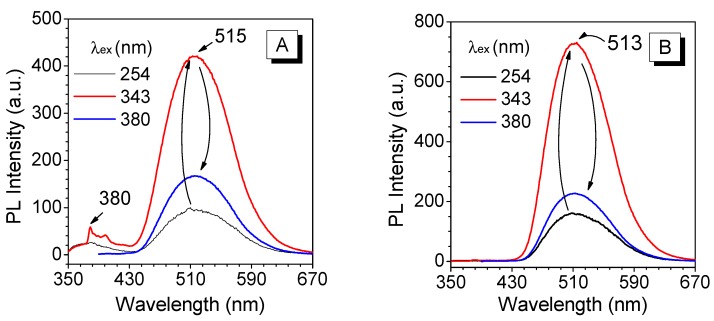
PL spectra of P**1** (**A**) and P**3** (**B**) in solution excited at different wavelengths: 254 nm (black), 343 nm (red) and 380 nm (blue). Solvent: THF, concentration: 0.02 mmol/L.

The existence of the energy transfer process can be confirmed by the time-resolved fluorescence spectroscope [42]. Without this instrument at hand, this related process has been further proved by the comparative study of the emission behaviors under different excitation wavelengths, considering that the emissions from different luminescent species are sensitive to different excitation wavelengths. As shown in Figure 6A, when the P**1** THF solution was excited with the UV-light source of 254 nm, the emission bands with peaks at 515 and 380 nm were recorded, and the intensity ratio of (*I*_515_/*I*_380_) is about 3.7. When the excitation wavelength changed to 380 nm, the emission band peaked at around 513 nm enhanced evidently but the emission band for the pyrene monomer vanished because only the poly(diphenylacetylene) backbone could absorb this excitation but the pyrene moieties could not. Under the excitation of 343 nm (Figure 6A,B), where the pyrene moieties have an intense absorption and the poly(diphenylacetylene) backbone has a moderate absorption, the PL spectrum shows the emission bands for the poly(diphenylacetylene) backbone (λ_em_~515 nm) and pyrene monomer (λ_em_~380 nm) accordingly. The ratio of *I*_515_/*I*_380_ is as high as 7.4, or twice of that recorded under the excitation of 254 nm UV-light, implying a net intensity gain for *I*_515_. Moreover, the absolute PL intensity for the poly(diphenylacetylene) backbone at the excitation wavelength of 343 nm is about 2.53 times of that recorded at the excitation wavelength of 380 nm. The net increase in the emission intensity can be associated with the contribution of the emission of pyrene moieties. In other words, there exists an energy transfer from the pyrene monomers to the poly(diphenylacetylene) backbone.

Another influential factor of the alkyl spacer between the pyrene and polymer backbone is the formation of the pyrene excimer. In our previous works of pyrene-modified mono-substituted polyacetylenes, we observed that both of the polymer chain and spacer have effects on the formation of pyrene excimers [36,37,38]. To evaluate the impact of the alkyl spacer on the excimer formation, we designed and synthesized polymer P**4**, an analogue of P**2**. The difference between P**4** and P**2** is the absence and presence of the alkyl spacer between the pyrene group and the poly(phenylacetylene) backbone. In the THF solution, the absorption features of P**4** and P**2** are identical (Figure 4A). However, the PL features demonstrate disparity in the emission band of the pyrene monomer and excimer. As revealed by the blue and olive spectral lines, the ratio of the emission intensity for the pyrene excimer (I_480_) and monomer (I_380_) in P**4** is about 1:0.47, but this value for P**2** is 1:1. These results suggest that the excimer species are more easily to be achieved in P**4** than in P**2**. This is reasonable because the polymer main chain pulls the pyrene groups together and allows to form excimers by nearby pyrene groups when the spacer is short. 

This is also true in the case of P**3** versus P**1**. As displayed in Figure 4B, there is almost no pyrene monomer’s emission for P**3** but there exists a weak but clear emission band from the pyrene monomers for P**1**, implying that, on one hand, the emission from the pyrene monomers is directly quenched by the energy transfer to the polymer backbone, and on the other hand, a part of the pyrene groups on P**3** readily form excimers due to the dragging effect of the poly(diphenylacetylene) backbone. The broadening of the proton peaks in the ^1^H NMR spectra of the polymers serve for good side proofs (Figure 3). If the intensity of the emission band (I_515_) of the poly(diphenylacetylene) backbone P**3** were compared under the excitation with different wavelengths (343 nm and 380 nm), it is found that the ratio is about 3.23, which is larger than that observed in the case of P**1** (2.53) (Figure 6A,B).

## 5. Conclusions

In summary, the electronic interactions between the pyrene groups on the side chains and the poly(diphenylacetylene) main chain have been investigated by using a series of intentionally designed pyrene-functionalized polyacetylenes. Based on the characteristic variation of the emissions from the pyrene monomer, pyrene excimer and poly(diphenylacetylene) backbone, the energy transfer from both of the pyrene monomers and excimers in the photo-excited states has been confirmed. This process dramatically and concomitantly quenched the pyrene monomer’s and excimer’s emission. For poly(phenylacetylene)s, the energy transfer is blocked due to the non-emissive polymer backbone thus the pyrene excimer’s emission dominated the photoluminescence spectrum. For poly(diphenylacetylenes), the fluorescence quenching also showed evident dependence on the alkyl spacer between the side pyrene group and the polymer backbone, which is associating with the co-existence of pyrene monomers and excimers. Considering that poly(diphenylacetylenes) are promising opto/electronic polymers, the understanding of the electronic interactions between the fluorescent polymer backbone and the modified functional groups is of great significance.

## Figures and Tables

**Table 1:** Polymer Reactions of P**0** and other precursors with pyrene-methylamine ^1^.

Entry	Polymer	Yield (%)	*M*_w_^2^	*M*_w_/*M*_n_^2^
1	P0	61.2	13,400	2.13
2	P1	94.1	12,100	2.10
3	P2	95.6	29,400	1.99
4	P3	89.2	65,200	3.14
5	P4	94.8	91,400	2.13

^1^ Carried out at room temperature under nitrogen for 12 h in THF/TEA, [P**0**]:[amine] = 1:1.1. P**1** was prepared from P**0**, while P**2**, P**3** and P**4** were prepared from precursors reported elsewhere. ^2^ Estimated by the gel permeation chromatograph in THF on the basis of polystyrene calibration.

## Supplementary Materials

The following are available online at https://www.mdpi.com/2073-4360/11/8/1366/s1. Experimental procedures and structure characterization data. Scheme S1, synthetic route to P**2**; Scheme S2, synthetic route to P**3**; Scheme S3, synthetic route to P**4**. Figure S1: Absorption spectra of M1 in the THF solution (0.02 mmol/L); Figure S2: Amplified PL spectrum showing the pyrene monomer emission band of P**3**. Figure S3: PL spectrum of pyrene-methylamine in the THF solution (0.02 mmol/L).
