# Supplementary material for: Pyrene-Functionalized Polyacetylenes: Synthesis and Photoluminescence Property
Source: Polymers (Basel). 2019 Aug 19;11(8):1366. doi: 10.3390/polym11081366 (PMC6723286; doi:10.3390/polym11081366)
Supplement: Supplementary file 1 [file polymers-11-01366-s001.pdf]

Supplementary Materials for

Pyrene-functionalized Polyacetylenes: Synthesis and Photoluminescence Property

Tanxiao Shen[1], Nan Jiang[1], Xiao'a Zhang[1], Lirong He[2], Xian Hua Lang[3], Jing Zhi Sun[1,*] and Hui Zhao[2,*]

1 MOE Key Laboratory of Macromolecular Synthesis and Functionalization, Department of Polymer Science and Engineering, Zhejiang University, Hangzhou 310027 China

2 Institute of Technical and Macromolecular Chemistry, University of Hamburg, Bundestrasse 45, 20146 Hamburg, Germany

3 Institute of Fundamental and Frontier Sciences, University of Electronic Science and Technology of China, 610051, Chengdu, China

* Correspondence: sunjz@zju.edu.cn; zhaoh@uestc.edu.cn.

Contents

Synthesis and characterization S2

Scheme S1 S5

Scheme S2 S 5

Scheme S3 S 6

Figure S1 S 6

Figure S2 S7

Figure S3 S7

Synthesis and characterization.

*Synthesis of monomer **1***. The synthetic route is demonstrated as Scheme 1 in the main text. The detailed experimental procedures are described as following.

*Methyl 6-(4-(phenylethynyl)phenoxy)hexanoate (**4**)*. Into a 250 mL round-bottom flask were placed 70 mg (0.1 mmol) $PdCl_2(PPh_3)_2$, 38 mg (0.2 mmol) CuI and 26 mg (0.1 mmol) PPh_3. After being evacuated and refilled with nitrogen for three times, 3.48 g compound **6** (10 mmol) in 30 mLTHF solvent and 40 mLtriethylamine and 20 mL piperidine. 2.2 mL (20 mmol) phenylacetylene was injected into the system after the catalyst was dissolved totally. The resulting mixture was stirred at room temperature for 24 h. After filtering, the solid was washed with diethyl ether, and the filtrate was concentrated by a rotary evaporator. The crude product was purified by a silica gel column using petroleum ether/ethyl acetate (v/v = 10:1) as eluent. A white solid was isolated in a yield of 84.2 %. ^{1}H NMR (400MHz, $CDCl_3$, δ, TMS, ppm): 7.53 (2H, d), 7.48 (2H, d), 7.30-7.36 (3H, m, 5), 6.87 (2H, d), 3.98 (2H, t), 3.68 (3H, s), 2.37 (2H, t), 1.77-1.84 (2H, m, 5), 1.67-1.75 (2H, m, 5), 1.48-1.54 (2H, m, 5). ^{13}C NMR (100MHz, $CDCl_3$, δ, TMS, ppm): 174.0, 159.0, 133.0, 131.4, 128.27, 127.9, 123.6, 115.1, 114.4, 89.4, 88.0, 67.6, 51.5, 33.9. FTIR, ν (cm^{-1}, in KBr pellet): 3054, 2997, 2947, 2866, 2216 (***C≡C***), 1780 (O***C=O***), 1604, 1565, 1513, 1467, 1438, 1386, 1287, 1249, 1166, 1106, 1030, 922, 880, 841, 805, 752, 688, 554, 518.

*6-(4-(Phenylethynyl)phenoxy)hexanoic acid (**3**)*. Into a 250 mL round-bottom flask were placed 2.93 g (9.1 mmol) compound **4**, 3.06 g (54.6 mmol) KOH, 50 mL THF and 50 mL methanol. The reaction system was refluxed for 8 h under stirring, then the resultant mixture was poured into 1000 mL 1 M HCl solution. The mixture was extracted with DCM for three times after it was cooled to room temperatrue, and the organic phase was dried with $MgSO_4$ powder. The solvent was evaporated by a rotary evaporator. The crude product was purified by a silica gel column using petroleum ether/ethyl acetate (v/v = 10:1) as eluent. A white solid was obtained in a yield of 92.6 %. ^{1}H NMR (400MHz, DMSO-d_6, δ, ppm): 7.55 (2H, d), 7.50 (2H, d), 7.31-7.39 (3H, m,5), 6.90 (2H, d), 4.00 (2H, t), 2.45 (2H, t), 1.99 (2H, m, 5), 2.04 (2H, m, 5), 2.01 (2H, m, 5). ^{13}C NMR (100MHz, DMSO-d_6, δ, ppm):180.2, 159.0, 133.0, 131.4, 128.3, 127.9, 123.6, 115.2, 114.5, 89.4, 88.0, 67.6, 33.9, 28.8. FTIR, ν (cm^{-1}, in KBr pellet): 3045, 2947, 2866, 2686, 2645, 2596, 2532, 2216 (***C≡C***), 1701 (***C=O***OH), 1598,

1510, 1470, 1419, 1291, 1252, 1205, 1174, 1137, 1108, 1073, 1037, 962, 936, 834, 801, 752, 688, 560, 524.

Perfluorophenyl 6-(4-(phenylethynyl)phenoxy)hexanoate (1). Compound **3** (1.66 g, 5.37 mmol), pentafluorophenol (0.99 g, 5.37 mmol), DCC (1.66 g, 8.06 mmol), DMAP (39.4 mg, 0.323 mmol), TsOH (61 mg, 0.323 mmol) and 150 mL dry DCM were added into a 250 mL round-bottom flask. The reacion mixture was stirring for 12 h at room temperature and then removed the solid with filtration. The liquid was concentrated by a rotatory evaporator, then the crude product was purified with a silica gel column using petroleum ether/ethyl acetate (v/v = 9:1) as eluent. A white solid was obtained in a yield of 86.8%。 ^{1}H NMR (400MHz, $CDCl_3$, δ, TMS, ppm): 7.50 (2H, d), 7.45 (2H, d), 7.29-7.35 (3H, m, 5), 6.86 (2H, d), 4.00 (2H, t), 2.69 (2H, t), 1.81-1.88 (4H, m, 5), 1.56-1.64 (2H, m, 5). ^{13}C NMR (100MHz, $CDCl_3$, δ, TMS, ppm): 169.3, 159.0, 142.3, 139.8, 139.2, 138.0, 136.6, 133.0, 131.4, 128.3, 127.9, 123.6, 115.3, 114.4, 89.4, 88.0, 67.5, 33.2, 28.7, 25.4, 24.5. FTIR, ν (cm^{-1}, in KBr pellet): 3436 (water), 3061, 2956, 2864, 2216 (***C≡C***), 1786 (O***C=O***), 1604, 1516, 1388, 1297, 1255, 1094, 994, 888, 830, 756, 693, 517.

*Synthesis of polymers P**0**, P**1**, P**2**, P**3** **and** P**4**.* All the polymerization reaction and post-polymerization modification reactions were carried out under nitrogen using Schlenk techniques in a vacuum-line system. Typical experimental procedures for the polymerization of monomer **1** catalyzed by WCl_6-Ph_4Sn complex are given below as an example. In a Schlenk tube were added 0.474 g monomer **1** and 3 mL toluene, and then the system was evacuated and refilled with nitrogen for three times. The catalyst of 20 mg WCl_6 and 21.6 mg Ph_4Sn dissolved in 2 mL toluene, the mixture was incubated for 15 min then injected into the Schlenk tube. The system was stirred 100 ^{o}C for 24 hours. Finally, the yellow resultant was precipitated in a 500 mL methanol, and the precipitate was collected. The product P**0** was obtained with a yield of 21.6% after drying to constant weight in vacuum oven. Characterization data: M_w 13400; M_w/M_n 2.13 (GPC, polystyrene calibration). ^{1}H NMR (500MHz, $CDCl_3$), δ (TMS, ppm): 6.59-7.08, 6.17, 3.82, 2.73, 1.83, 1.60. ^{13}C NMR (125MHz, $CDCl_3$), δ (TMS, ppm): 169.3, 156.6, 145.2, 142.2, 140.6, 139.7, 138.9, 138.0, 136.5, 131.8, 130.7, 129.3, 129.1, 128.4, 127.2, 126.0, 125.0, 67.5, 33.1, 28.8, 25.4, 24.4. FTIR, ν (cm^{-1}, in KBr pellet): 3429 (water), 3052, 2938, 2864, 1786 (O***C=O***), 1607, 1519, 1470, 1284, 1242, 1173, 1082, 1002, 821, 752, 692, 553.

*Synthesis of P**1**.* After 59.3 mg (0.125 mmol) P**0** was added into a 10 mL Schlenk tube, the system was evacuated and refilled with nitrogen for three times. Then, 4 mL THF, 3 drops of dried

triethylamine, and 31.9 mg (0.138 mmol) pyrene-methylamine were introduced into the reaction system. Stirring at room temperature for 12 h, the resultant mixture was added into 200 mL methanol under violent stirring through cotton filter. After standing still for 24 h, the precipitate was collected by filtration, washed with methanol for three times and dried in air. 21.6% after drying to constant weight in vacuum oven. The yield of P**1** was 94.1%. Characterization data: M_w 12100; M_w/M_n 2.10 (GPC, polystyrene calibration). ^{1}H NMR (500 MHz, $CDCl_3$), δ (TMS, ppm): 8.47, 8.31, 8.14, 8.02, 7.05, 6.62, 6.18, 4.93, 3.67, 2.10, 1.48, 1.24. ^{13}C NMR (125 MHz, $CDCl_3$), δ (TMS, ppm): 172.3, 133.3, 131.0, 130.6, 130.4, 128.4, 127.7, 127.3, 126.4, 125.4, 124.9, 124.3, 124.2, 123.5. FTIR, ν (cm^{-1}, in KBr pellet): 3323 and 3306 (***NH***C=O), 3038, 2940, 2863, 1655 (NH***C=O***), 1603, 1508, 1468, 1440, 1417, 1389, 1282, 1245, 1175, 1109, 1068, 1030, 969, 905, 844, 816, 758, 694, 620, 546.

The synthesis procedures of P**2**, P**3**, and P**4** were similar to that of P**1**. The synthetic route was shown in Scheme S1, S2 and S3, respectively. The characterization data are as following. P**2**: red solid, yield 95.6%. M_w 91400; M_w/M_n 1.99 (GPC, polystyrene calibration). FTIR, ν (cm^{-1}, in KBr pellet): 3324 and 3306 (***NH***C=O), 3038, 2940, 2863, 1656 (NH***C=O***), 1603, 1530, 1493, 1289, 1186, 1146, 1107, 842, 763, 707, 623, 553. P**3**: yelllow solid, yield 89.2%. M_w 65200; M_w/M_n 3.14 (GPC, polystyrene calibration). FTIR, ν (cm^{-1}, in KBr pellet): 3423 and 3306 (***NH***C=O), 3040, 2922, 1648 (NH***C=O***), 1604, 1530, 1495, 1290, 1184, 1144, 1106, 843, 765, 705, 624, 552. P**4**: orange solid, yield 94.8%; M_w 28700; M_w/M_n 2.05 (GPC, polystyrene calibration). FTIR, ν (cm^{-1}, in KBr pellet): 3423 and 3306 (***NH***C=O), 3040, 2922, 1646 (NH***C=O***), 1603, 1530, 1493, 1289, 1186, 1146, 1107, 842, 763, 707, 623, 553.

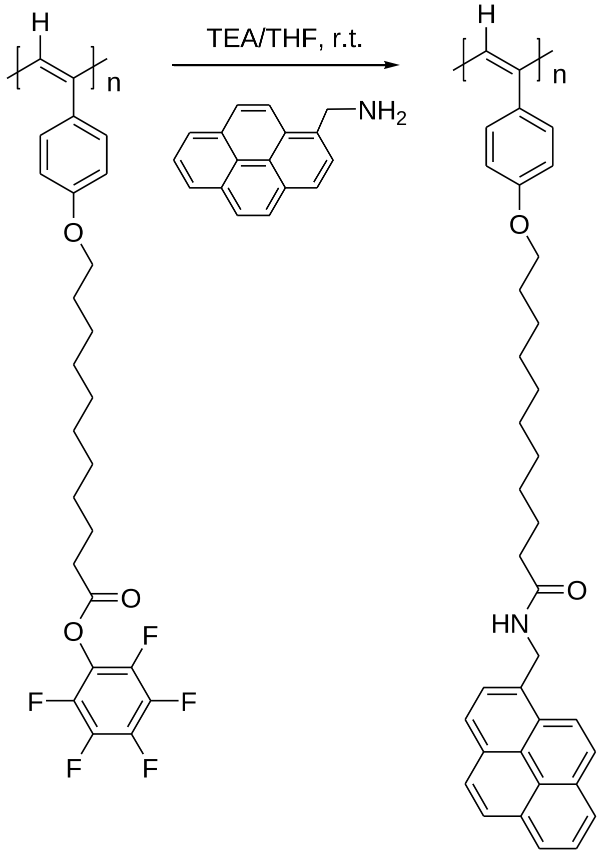

P**2**

Scheme S1. Synthetic route to P**2**.

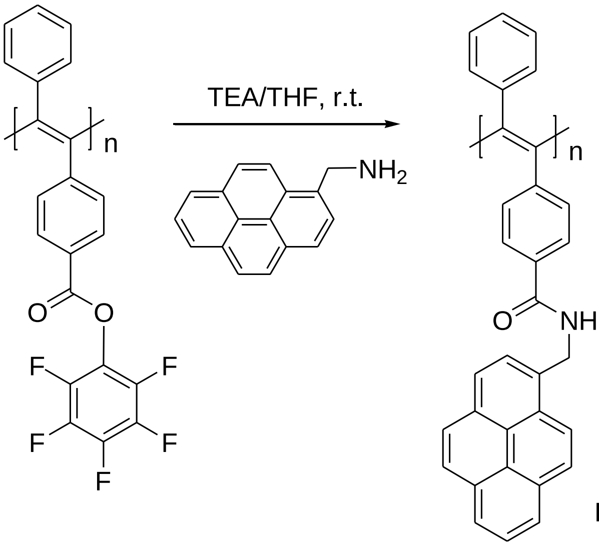

P**3**

Scheme S2. Synthetic route to P**3**.

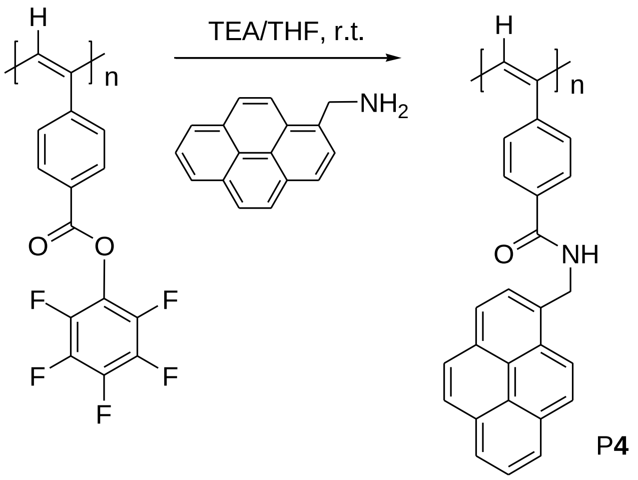

Scheme S3. Synthetic route to P**4**.

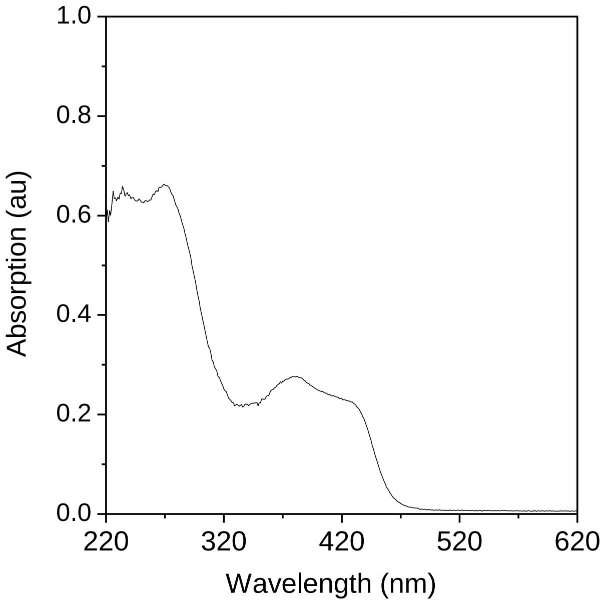

Figure S1. Absorption spectrum of M1 in THFsolution. Concentration: 0.02 mmol/L.

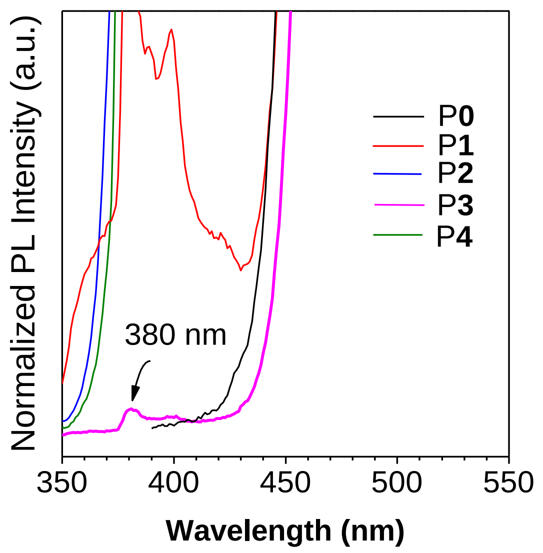

Figure S2. Amplified PL spectrum showing the pyrene monomer emission band of P**3**.

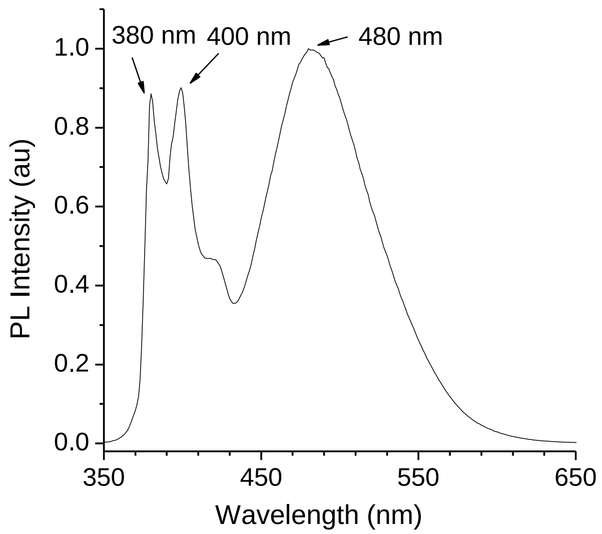

Figure S3. PL spectrum of pyrene-methylamine in THF solution (0.02 mmol/L).
